# High sedentary time in children is not only due to screen media use: a cross-sectional study

**DOI:** 10.1186/s12887-019-1521-8

**Published:** 2019-05-16

**Authors:** Belinda Hoffmann, Susanne Kobel, Olivia Wartha, Sarah Kettner, Jens Dreyhaupt, Jürgen M. Steinacker

**Affiliations:** 1 Division of Sports- and Rehabilitation Medicine, Center of Medicine, Ulm University Hospital, Leimgrubenweg 14, 89075 Ulm, Germany; 2 Institute of Epidemiology and Medical Biometry, Ulm University, Schwabstraße 13, 89075 Ulm, Germany

**Keywords:** Sedentary lifestyle, Public health, Screen time, Primary school

## Abstract

**Background:**

Sedentary behaviour has become a growing public health concern. Currently, it is a common belief that screen time (SCT) is a key factor in high overall sedentary time (ST) and is often used as a primary outcome. However, the evidence is lacking. Therefore, this study investigated the association of objectively assessed total ST with SCT among children. Further, SCT was investigated separately for sedentary level, weight status, gender, and migration background.

**Methods:**

For 198 primary school children (7.1 ± 0.7 years, boys: 43.9%) ST was assessed objectively using a multi-sensor device (Actiheart®; CamNtech, Cambridge, UK). The sample was split into three groups (tertiles) to investigate SCT of children with low, medium and high ST. SCT and socio-demographic parameters, such as migration background, were assessed using a parental questionnaire; anthropometric data was collected at schools.

**Results:**

Absolut SCT did not differ significantly among the three sedentary groups: Daily average of SCT was 83.8 ± 55.0 min (27.4% of ST) for children with high ST, 82.8 ± 50.5 min (39.8% of ST) for children with medium ST, and 77.2 ± 59.4 min (71.3% of ST) for those with low ST. However, relatively the SCT percentage of total ST was significantly higher among children with low ST (*p* < 0.01). Significantly higher SCT was found in children with migration background (p < 0.01), while underweight children had significantly less SCT (*p* < 0.05). An association of total SCT and overall ST was found for the whole sample (B = 17.11, [2.75; 31.48], *p* = 0.02), but did not remain when analysis were separated for the groups, except for normal weight children (B = 15.97, [0.13; 31.81], *p* = 0.05).

**Conclusions:**

The amount of SCT is the same among high, low and medium sedentary children, and high ST is largely independent of SCT. Therefore, SCT cannot be the key contributor to high ST and should not solely be used for predicting or changing children’s sedentary behaviour. Moreover, children’s weight status to classify activity levels and the role of possible compensation mechanisms should be considered in future research and when trying to intervene on ST.

**Trial registration:**

German Clinical Trials Register (DRKS), DRKS-ID: DRKS00000494 DATE: 25/08/2010.

## Introduction

Sedentary behaviour has become a growing public health concern, especially since it has been identified to be a risk factor for health in youth [1] regardless of physical activity patterns [2, 3]. Further, sedentary behaviour can have impacts into adulthood including the risk of suffering from its associated negative health consequences in later life [4, 5]. Even though sedentary behaviour has been suggested to be an independent health risk factor, it is widespread. The amount of sedentary time (ST) in zero to 12-year-old European children ranges from 3.2 to 9.2 h a day [6]. Children spend up to half of their after-school period with sedentary behaviours (41–51%; 5–12 years), a number that increases with adolescence (57%; 12–18 years) [7].

Most of the associated risk factors were identified when screen media use was assessed [8]. Research shows that elevated screen media use is a risk for health, as it has been associated with most of the previously investigated health aspects [1], especially with obesity [1, 8, 9]. Therefore, it has been recommended to limit screen media use for children to a maximum of two hours daily [10]; in Germany, however, for primary school children a limit of one hour per day was suggested [11]. Similar to overall sedentariness, there is a high prevalence of high screen time (SCT) in children already [3, 6]. It has been shown that European children spend up to 2.7 h watching TV per day [6].

There is a common belief that screen media use and sedentariness are associated with another. However, studies suggest that neither self-reported SCT represents overall ST adequately [12–14], nor that a relation of SCT and total ST exists [15]. Therefore, SCT should be investigated as one part of ST. So far, only one current review reported percentages of watching television (12.6–31.0%) or screen-based sedentary behaviour (8.5–25.3%) of children’s ST [7]. However, these percentages refer only to the after-school period and the result is based on different studies using different assessments. This probably caused the relatively wide range in percentages.

Still, SCT is used as a proxy for ST in most studies; however, media use is not representing overall ST [12–14] and hence might be an incorrect measure of assessment. Even if ST is assessed objectively, it is unclear what proportion of ST is based on screen media use. Therefore, this study aimed to investigate the proportion of SCT on objectively assessed overall ST, as well as their association among German primary school children with high, medium, and low levels of ST. Moreover, the study targeted to examine the SCT proportion of ST and the association with weight status, migration background and gender.

## Methods

This study aimed to investigate the association of SCT and ST among German primary school children, as well as the SCT proportion of objectively assessed overall ST. Further, the proportion and association were examined separately for children with high, medium, and low levels of ST, migration background, gender and weight status.

### Participants

Data of the cross-sectional ‘Baden-Württemberg Study’ was analysed (registered at the German Clinical Trials Register [DRKS-ID DRKS00000494]) [16, 17]. Within this study, in a sub-sample of 384 primary school children, physical activity and ST were assessed objectively. Valid data for ST and SCT was available from 198 children. The children were 7.1 ± 0.7 years on average and 43.9% were male. All characteristics of the sample are shown in Table 1.

**Table 1:** Characteristics of the sample and separated for sedentary levels

	Total		Low ST		Medium ST		High ST		Significance
	n	N (%)	n	N (%)	n	N (%)	n	N (%)	
Boys	198	87 (43.9)	66	38 (57.6)	66	30 (45.5)	66	19 (28.8)	p = 0.00**
		Mean (SD)		Mean (SD)		Mean (SD)		Mean (SD)	
Age (years)	198	7.1 (0.7)	66	7.1 (0.7)	66	7.2 (0.7)	66	7.0 (0.6)	*p =* 0.20
Height (cm)	198	124.0 (6.0)	66	124.5 (6.3)	66	124.9 (6.6)	66	122.5 (4.8)	p = 0.05*
Weight (kg)	198	24.6 (4.9)	66	25.1 (5.7)	66	25.5 (5.1)	66	23.3 (3.1)	*p =* 0.02*
BMI	198	15.9 (2.2)	66	16.0 (2.3)	66	16.3 (2.6)	66	15.5 (1.5)	*p =* 0.13
BMIPCT^a^	198	46.9 (26.8)	66	46.9 (28.5)	66	50.4 (27.7)	66	43.4 (24.0)	*p =* 0.33
Weight status^b^		N (%)		N (%)		N (%)		N (%)	
Underweight	198	13 (6.6)	66	6 (9.1)	66	2 (3.0)	66	5 (7.6)	*p =* 0.37
Normalweight	198	168 (84.8)	66	52 (78.8)	66	58 (87.9)	66	58 (87.9)	*p =* 0.81
Overweight/Obese	198	17 (8.6)	66	8 (12.1)	66	6 (9.1)	66	3 (4.5)	*p =* 0.33
Migration background^c^	195	48 (24.6)	66	10 (15.2)	64	18 (28.1)	65	20 (30.8)	*p =* 0.09
Screen time		Mean (SD)		Mean (SD)		Mean (SD)		Mean (SD)	
Total (min/day)	198	81.3 (54.9)	66	77.2 (59.4)	66	82.8 (50.5)	66	83.8 (55.0)	*p =* 0.14
Weekday (min/day)	198	61.9 (51.5)	66	58.6 (54.5)	66	62.3 (47.2)	66	64.8 (53.1)	*p =* 0.45
Weekend (min/day)	198	129.7 (77.1)	66	123.6 (80.8)	66	134.1 (75.4)	66	131.4 (75.9)	*p =* 0.34
Reaching screen-media guideline		N (%)		N (%)		N (%)		N (%)	
≤ 1 h/day^d^	198	84 (42.4)	66	28 (42.4)	66	28 (42.4)	66	28 (42.4)	*p =* 1.00
≤ 2 h/day^e^	198	166 (83.8)	66	57 (86.4)	66	56 (84.8)	66	53 (80.3)	*p =* 0.62
Percentage of screen time of ST^f^		Mean (SD)		Mean (SD)		Mean (SD)		Mean (SD)	
Total (min/day)	198	46.2 (41.4)	66	71.3 (56.3)	66	39.8 (26.1)	66	27.4 (17.7)	p = 0.00**
Weekday (min/day)	198	39.6 (43.6)	66	64.5 (60.0)	66	31.6 (28.6)	66	22.8 (18.9)	p = 0.01*
Weekend (min/day)	198	67.9 (60.0)	66	98.6 (80.6)	66	65.6 (45.5)	66	39.5 (23.3)	p = 0.00**
Sedentary time		Mean (SD)		Mean (SD)		Mean (SD)		Mean (SD)	
Total (min/day)	198	212.6 (87.7)	66	116.4 (35.6)	66	212.5 (26.9)	66	309.0 (49.7)	p = 0.00**
Weekday (min/day)	198	201.7 (92.8)	66	102.1 (40.5)	66	207.0 (36.3)	66	295.9 (63.2)	p = 0.00**
Weekend (min/day)	198	240.0 (105.4)	66	152.1 (65.4)	66	226.2 (68.3)	66	341.6 (78.7)	p = 0.00**
Recording times (min/day)	198	1424.9 (34.1)	66	1430.9 (29.7)	66	1421.7 (38.7)	66	1422.0 (32.9)	*p =* 0.22

**significant (*p* < 0.01); *significant (*p* < 0.05); ^a^Body mass index percentiles on the basis of Kromeyer-Hausschild et al., 2001; ^b^classifiyed BMI percentiles (BMIPCT) on the basis of Kromeyer-Hausschild et al., 2001; ^c^defined as having at least one parent who was born abroad or having a parent who spoke to their child in a foreign language during the first three years of their life; ^d^according to Rütten & Pfeifer, 2016; ^e^according to Tremblay et al., 2016; ^f^calculated screen time percentages of total sedentary time (=ST) based on the averages of each child

### Assessment of ST

ST was assessed using a validated multi-sensor device (Actiheart®, CamNtech, Cambridge, the UK) which was fitted to the child’s chest at school and was worn for six consecutive days à 24 h. The recordings had to include a minimum of 10 h per day on at least one day of the weekend and two weekdays [18]. 15-s epochs were used to record one-dimensional bodily acceleration in counts per minute (cpm) and heart rate (bpm). Energy expenditure was calculated as metabolic equivalents (MET) and total ST was defined standardly (ST ≤ 1.5 MET) [19]. Children’s ST was calculated individually without sleep (for more details see [20]). Valid data of individual ST was available for 231 children. To classify children into groups of low, medium and high ST, the sample was split into thirds with the same sample size (tertiles): 66 children = low ST (≤ 165 min), 66 children = medium ST (> 165 min ≤ 251 min), 66 children = high ST (> 251 min).

### Assessment of SCT

SCT was assessed using a well-established and validated parental questionnaire [21]. Parents were asked how much daily time their child spends watching television or videos (TV) and playing PC or console games (PC) for weekdays and weekends separately. Answers were given in categories (never, up to 0.5 h, 0.5-1 h, 1-2 h, 2-3 h, 3-4 h, > 4 h). In each category, the upper limit was used and daily total SCT was calculated for each child as follows: mean total SCT = [(TV + PC weekday × 5) + (TV + PC weekend day × 2)]/7. If children exceeded national (SCT ≤ 1 h) [11] and international guidelines of SCT (SCT ≤ 2 h) [10] were investigated separately. Sufficient valid data on SCT was available for 198 children.

### Assessment of child-related factors

Child-related factors such as gender, age, and migration background were collected using the parental questionnaire. For migration background, children had to have at least one parent who was born abroad or were spoken to in a foreign language during the first three years of their life. To calculate children’s BMI (kg/m^2^), anthropometric data was assessed at schools by trained staff. Weight was measured to the nearest 0.05 kg using weighing scales and height to the nearest 0.1 cm using a stadiometer (Seca 862 and Seca 213, respectively, Seca Weighing and Measuring Systems Hamburg, Germany). For children’s weight status, BMI percentiles (BMIPCT) were calculated according to German reference data [22]. Children were categorised into underweight (≤10 percentile), normal weight (> 10 to ≤90 percentile), and overweight and/or obese (> 90 percentile) [22].

### Statistical analysis

Participants’ characteristics were investigated descriptively. Amount of ST and SCT as well as the SCT percentage of ST (means, standard deviations) were calculated. Tests of normal distribution were performed for all variables. Screen time and SCT percent of ST weren’t distributed normally. To achieve normal distribution these two variables were logarithmised. Differences in relation to the main study sample as well as for the investigated groups (ST level, weight status, migration background, gender) were examined. For this, chi-square tests for categorial variables, and two-sample t-tests and analysis of variance (ANOVA) for continuous variables were performed. In the case of significant ANOVA results, the Bonferroni test was used to investigate pairwise group differences. Paired sample t-tests were used to detect differences between SCT on weekdays and at weekends. Pearson’s correlation coefficients and a multiple linear regression model (adjusted for gender, weight, and height) were performed to investigate associations of ST and SCT. For statistical analysis, SPSS Statistics 25 (IBM Corp. Armonk, NY, USA) was used. The level of significance was set to *p* ≤ 0.05, two-sided.

## Results

The analysed sample did not differ from the main study sample, except for gender (girls: + 8.0%; *p* < 0.05) and migration background (− 8.7%; p < 0.05). On average, children’s total ST was 212.6 ± 87.7 min per day. Screen media was used for 81.3 ± 54.9 min daily. The average percentage of SCT was 46.2 ± 41.4% of their total ST. The guideline of no more than 1 h SCT per day was reached by 42.4% (*n* = 84) of children and a maximum of 2 h by 83.8% (*n* = 166). Compared to weekdays, at the weekend, SCT and the SCT percentage of ST were significantly higher in the total sample as well as in all investigated groups (high, medium, and low ST level; separated for weight status, migration background, and gender; all *p* < 0.01). In the whole sample, neither a significant association of ST with SCT in total (r = 0.13; *p* = 0.06) nor for the weekend (r = 0.13; *p* = 0.07) or weekdays (r = 0.03; *p* = 0.68) was found. A significant association was found with the adjusted linear regression model for total ST with total SCT only (B = 17.11, [2.75; 31.48], *p* = 0.02), but not for weekday or weekend independently. When the association was assessed separately for the investigated groups the significance did remain among the normal weight children for total ST (B = 15.97, [0.13; 31.81], *p* = 0.05), and for ST on the weekend (B = 17.94, [0.23; 35.66], p = 0.05) with SCT, but not for weekdays ST and SCT. Among all other investigated groups, no significant associations of SCT with ST were found; neither in total nor separated for weekdays or weekends.

### Sedentary levels and screen time

**Fig. 1:**
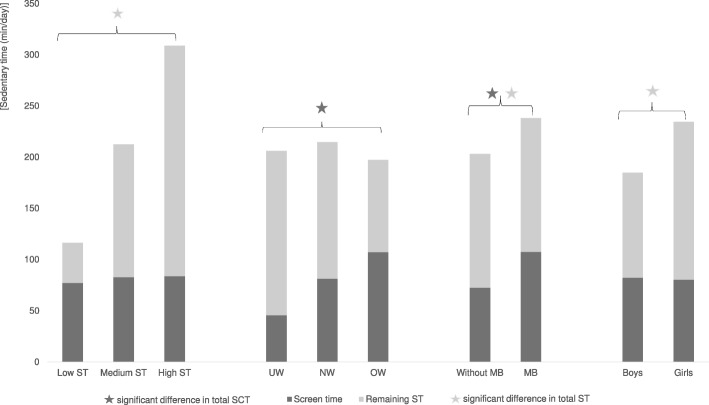
Daily amount of SCT on ST, separated for sedentary level, weight status, migration background, and gender. *ST* sedentary time, *UW* underweight, *NW* normal weight, *OW* overweight/obese, *MB* migration background, *SCT* screen time

Characteristics of children in high, medium and low ST groups differed significantly for gender, height, and weight (s. Table 1). The daily average of SCT for children with high ST was 83.8 ± 55.0 min (27.4% of ST), for children in the middle tertile it was 82.8 ± 50.5 min (39.8% of ST), and of those with low ST 77.2 ± 59.4 min (71.3% of ST). No significant group differences were found for SCT (total: F = 1.98, *p* = 0.14; week: F = 0.81, *p* = 0.45; weekend: F = 1.08, *p* = 0.34), which is shown in Fig. 1. Overall ST (F = 411.81, *p* < 0.01) and the overall SCT percentage of ST (F = 13.44; p < 0.01), as well as the percentage on weekdays (F = 4.74, *p* = 0.01) and on the weekend (F = 9.82, p < 0.01) were significantly different among the three groups. For both media use guidelines (SCT ≤ 1 h, SCT ≤ 2 h) no significant group differences were found.

### Weight status and screen time

Daily ST and SCT of the three weight status groups are shown in Fig. 1. Significant group differences were found for SCT (total: F = 6.86, *p* = 0.00; week: F = 5.67, p = 0.00; weekend: F = 4.47 p = 0.01) and for SCT percentages (total: F = 5.91, *p* = 0.01; week: F = 5.21, p = 0.01; weekend: F = 4.77, p = 0.01), but not for ST.

As seen in Table 2, among obese and/or overweight children screen media use on average covers 69.5% of their daily overall ST (107.4 ± 58.5 min). Normal weight children spent 45.3% of their overall ST with SCT (81.4 ± 54.5 min) and underweight children accumulated 27.5% of SCT per day (45.8 ± 34.1 min). In comparison to their normal weight counterparts (Table 2), SCT of obese and/or overweight children did not differ significantly. Percentage of SCT at the weekend was found to differ significantly (*p* < 0.05), but not on weekdays or in total. Both SCT guidelines were exceeded significantly more often by obese and/or overweight children (*p* < 0.01). Underweight children reached both SCT recommendations significantly more often (p < 0.01) compared to normal weight children. SCT and percentage of SCT were significantly lower in the group of underweight children (*p* < 0.05), except for the weekend (SCT: *p* = 0.06; PCT: *p* = 0.38).

**Table 2:** Daily ST, SCT and SCT percentages in total, on weekdays and at the weekend separated for weight status, migration background, and gender

Weight status^1^	Normal weight^2^			Overweight/Obese^3^			Significance	
	n	Mean (SD)	% of ST	n	Mean (SD)	% of ST	Mean	% of ST
Total ST	168	214.6 (86.3)	–	17	197.5 (88.4)	–	p = 1.00	–
Total SCT	168	81.4 (54.5)	45.3	17	107.4 (58.5)	69.5	*p* = 0.19	*p* = 0.11
Weekday SCT	168	61.9 (50.7)	39.3	17	86.1 (57.5)	57.4	*p* = 0.48	*p* = 0.46
Weekend SCT	168	130.0 (77.8)	64.1	17	160.6 (72.7)^a^	115.3	*p* = 0.32	p = 0.04*
SCT <1h^b^	70	–	41.7	5	–	29.4	–	p = 0.00**
SCT < 2h^c^	142	–	84.5	11	–	64.7	–	p = 0.00**
Weight status^1^	Normal weight^2^			Underweight^4^			Significance	
	n	Mean (SD)	% of ST	n	Mean (SD)	% of ST	Mean	% of ST
Total ST	168	214.6 (86.3)	–	13	206.1 (108.5)	–	p = 1.00	–
Total SCT	168	81.4 (54.5)	45.3	13	45.8 (34.1)	27.5	p = 0.01*	p = 0.04*
Weekday SCT	168	61.9 (50.7)	39.3	13	30.0 (36.7)	19.6	p = 0.01*	p = 0.02*
Weekend SCT	168	130.0 (77.8)	64.1	13	85.4 (53.2)^a^	54.3	p = 0.06	p = 0.38
SCT <1h^b^	70	–	41.7	9	–	69.2	–	p = 0.00**
SCT < 2h^c^	142	–	84.5	13	–	100	–	p = 0.00**
Migration background	Migration background^5^			No migration background			Significance	
	n	Mean (SD)	% of ST	n	Mean (SD)	% of ST	Mean	% of ST
Total ST	48	238.1 (79.3)	–	147	203.3 (88.3)	–	p = 0.02*	–
Total SCT	48	107.6 (57.9)	51.0	147	72.7 (51.7)	44.9	p = 0.00**	p = 0.06
Weekday SCT	48	82.8 (57.3)	43.8	147	55.3 (48.2)	38.6	*p* = 0.08	*p* = 0.25
Weekend SCT	48	169.4 (75.6)^a^	69.2	147	116.3 (73.9)	67.1	p = 0.00**	p = 0.02*
SCT <1h^b^	12	–	25.0	71	–	48.3	–	p = 0.00**
SCT < 2h^c^	31	–	64.6	132	–	89.9	–	p = 0.00**
Gender	Boys			Girls			Significance	
	n	Mean (SD)	% of ST	n	Mean (SD)	% of ST	Mean	% of ST
Total ST	87	184.8 (78.4)	–	111	234.4 (88.7)	–	p = 0.00**	–
Total SCT	87	82.5 (49.3)	53.2	111	80.3 (59.0)	40.7	*p* = 0.57	p = 0.02*
Weekday SCT	87	63.7 (45.8)	45.4	111	60.5 (55.7)	35.1	*p* = 0.27	p = 0.06
Weekend SCT	87	129.3 (73.5)^a^	78.3	111	130.0 (80.1)	59.7	*p* = 0.73	p = 0.07
SCT <1h^b^	31	–	35.6	53	–	47.7	–	p = 0.02*
SCT < 2h^c^	74	–	85.1	92	–	82.9	–	*p* = 0.16

**significant (*p* < 0.01); *significant (*p* < 0.05); ^a^significantly more SCT at the weekend (p < 0.01); ^b^according to to Rütten & Pfeifer, 2016; ^c^according to Tremblay et al., 2016; ^1^Body mass index percentiles by Kromeyer-Hausschild et al., 2001; ^2^ > 10 ≤ 90 body mass index percentiles on the basis of Kromeyer-Hausschild et al., 2001; ^3^ > 90 body mass index percentiles on the basis of Kromeyer-Hausschild et al., 2001; ^4^ ≤ 10 body mass index percentiles on the basis of Kromeyer-Hausschild et al., 2001; ^5^defined as having at least one parent who was born abroad or having a parent who spoke to their child in a foreign language during the first three years of their life

### Migration status and screen time

As seen in Fig. 1, total SCT and total ST were significantly higher in children with migration background, than in children without migration background (p < 0.05). Higher SCT among children with migration background was also found at the weekend (*p* < 0.01) (s. Table 2). SCT percentages of ST did not differ significantly, excepted from weekends percentage (*p* < 0.05). On average, children with migration background spent 107.6 ± 57.9 min per day using screen media, which corresponds to an average of 51.0% of their daily ST. Both guidelines for SCT were reached significantly less often by children with migration background (*p* < 0.01).

### Gender and screen time

Boys spent 82.5 ± 49.3 min and girls spent 80.3 ± 59.0 min per day using screen media with no significant difference of SCT (Fig. 1). Boys’ percentage of SCT of ST was significantly higher than in girls (Table2). The national recommendation of less than 1 h SCT was reached significantly more often by boys (p < 0.05), while for the international ≤2 h recommendation no significant gender difference was found. Overall ST among girls was significantly higher (p < 0.01). Furthermore, in the adjusted regression model of the whole sample gender was significantly associated with ST (B = 49.40 [25.86; 72.94], *p* = 0.00).

## Discussion

This study analysed SCT in a sample of German primary school children among objectively assessed high, medium and low ST levels and the association of SCT with total ST. On average, the whole sample spent 81 min daily using screen media, which corresponds to almost half (46.2%) of their total ST. Similar, 95 min of daily SCT among 6 to 7-year-old German children was described previously, also using parental report [23]. In a study by Tanaka et al. [15] 60% of children reached the current guideline of no more than 2 h SCT per day [10]. In contrast, in this study approximately 25% more children met this guideline (84%, *n* = 166), pointing to a lower amount if SCT is internationally compared. However, the German recommendation for primary school children of ≤1 h was reached by less than half of this sample (42%, *n* = 84). Furthermore, the results point towards no association of ST and SCT, as none of the correlations reached significance. Even though overall ST was found to be associated with SCT in total, neither the separate analysis of the weekdays and the weekend, nor of the investigated groups remained significant, except the group of normal weight children. Therefore, the overall results of this study indicate that ST is largely independent of SCT among primary school children of south-west Germany. This can be supported by the study of Tanka et al. (2017), where also no association of ST and SCT was found. Moreover, studies showed that SCT is not a proxy for overall ST [12–14].

### Sedentary levels and SCT

These results indicate that children’s large amounts of sedentary behaviour are not necessarily due to high media use. Neither SCT nor reaching either SCT guidelines differed among children with low, medium and high ST. Children in the highest ST group, i.e. those who spend more than 5 h being sedentary a day, spent the same amount of time with screen media as children with low ST (ø ST = 2 h), and those in the middle tertile (ø ST = 3.5 h), as seen in Fig. 1. Rather, the low sedentary group spent the largest part of their ST with SCT. Their SCT percentage of ST is more than twice as high (71%) as among the children with high ST (27%), in which the SCT percentage of ST was the lowest. This indicates that SCT forms the main part of sedentary behaviour among children with low ST, but not among children with high ST. So far, no previous research examined the percentage of SCT of overall ST among different ST levels. From one previous Japanese study [15] the percentage of SCT of ST could be calculated and was 40%, which is similar to the percentage of this sample (46%). However, those children were about two years older (9.3 years). Therefore, the percentage of those Japanese children would be expected to be higher, as screen media use increases with age [24, 25]. On the other hand, the questionnaire was completed jointly by the children and their parents, which could explain the variance as well [6]. However, SCT might not be the main behaviour among high sedentary children. This can be supported by the result that neither a correlation was found between SCT and ST in the whole sample, nor an association for any of these three groups. Similar, no association of subjectively assessed SCT and objectively measured ST was reported in a study with 426 primary school children [15].

### Weight status and SCT

The three weight status groups differed in SCT and percentage, but they did not differ in overall ST. The separated comparison to normal weight children showed that underweight children spent less than half of the time with SCT (30 min vs. 60 min). Moreover, all underweight children had less than 2 h of SCT and more underweight children met both SCT guidelines. In contrast, SCT and percentages of overweight and/or obese children did not differ significantly from normal weight children, except from the percentage on weekends. This higher percentage of SCT of ST and reaching both SCT guidelines less often could indicate that overweight and/or obese children might have higher amounts of SCT than normal weight children. But the percentage of SCT of ST at weekends was found to be more than 100%, which shows that the SCT at weekends of obese and/or overweight children might have been overestimated by their parents. However, because this was found only among overweight and obese children, the calculation of energy expenditure in metabolic equivalents could be a more likely reason. As children with higher fat mass have to carry more weight at the same work-load, they can be misclassified as more active [26]. These findings suggest that further research investigating SCT and ST especially among overweight and obese children is necessary. But it can be followed, that in this study only underweight children had lower amounts of SCT, while media use of obese and/or overweight children did not differ significantly in comparison to normal weight children. Only due to the explained aspects, the results can be interpreted as a possibility of higher SCT among obese and/or overweight children.

### Migration status and SCT

Confirming previous findings [27, 28], in this study, children with migration background spent more time using screen media than children without migration background. Especially on the weekend, almost one more hour SCT was observed and both guidelines of SCT were exceeded more often among children with migration background. Consequently, higher amounts of ST are very likely to be influenced by higher media use among children with migration background. However, this could not be supported by the regression models within this study. Therefore, the association needs to be analysed further, probably with larger sample sizes.

### Gender and SCT

As boys prefer playing computer or game console more often than girls during leisure time [23], it would have been expected that boys spent more time with screen media. Supporting this, fewer boys exceeded the guideline of no more than one hour SCT daily. However, there was no difference in SCT between boys and girls. On the other hand, gender was the only significant factor for ST in the regression model of this study and fewer boys were in the high sedentary group compared to the low sedentary group. Previous studies also reported an association between gender and ST [20, 24, 25]. That findings indicate that gender plays a greater role in children’s ST than in SCT. Consequently, the higher SCT percentage is likely a result of higher amounts of ST in girls and not due to higher media use of boys.

### Implications

Since ST in this sample is largely independent of SCT it is unlikely that common interventions decrease ST using a reduction of screen media use. This has been shown previously as interventions often only reach small effect sizes and that the mechanism of reducing ST still remain [29]. Many interventions aiming to reduce ST focus on behavioural change by increasing physical activity – often by adding sports [30]. However, it has been shown that children (and other populations, such as elite athletes) who are active are also more sedentary [31, 32]; i.e. they compensate LPA with ST [15]. This may be attributable to either behavioural aspects, such as preferences for sedentary activities, or the need for a physical rest after high intensity or long lasting activities. This needs to be considered when trying to reduce ST. Therefore, it might be more effective to change surroundings, offer attractive leisure time alternatives and increase day to day habitual (low intensity) physical activity instead of vigorous intensity exercise or sports to reduce ST. Some interventions have already adopted this approach [16, 33], however, further research is still necessary.

### Strengths and limitations

This is the first study investigating the proportion of subjectively assessed SCT of objectively assessed total ST and its contribution to high ST behaviour among a large cohort of primary school children of south-west Germany. The objective and individual assessment of children’s ST and anthropometric data are the main strengths of this study. However, some limitations need to be considered when interpreting these findings. The comparison of differently assessed data could have caused variations in the results, but an objective assessment of SCT was not feasible. Moreover, both SCT and ST might be over- or underestimated due to social desirability. Also proxy and recall probably cause variations in subjective assessed data. Even though ST was objectively assessed, it is not known what kind of sedentary activities the children really carried out during their sedentary periods. As SCT is only a small part of this “black box” a deeper insight into other sedentary activities other than SCT is necessary [34]. Therefore, the proportion of further sedentary activities of overall ST needs to be examined, especially among children with high ST. Nevertheless, this study analysed the objective data of ST in a large sample size.

## Conclusion

The main finding of this study indicates that SCT does not predict high ST in primary school children. Rather, high ST is largely independent of SCT. Even though SCT covers up to 71% of ST, children with high sedentary levels did not have higher SCT than those with low sedentary levels, except for children with migration background. Therefore, SCT does not seem to be the key contributor for high ST in children and cannot solely be used for predicting or changing children’s overall sedentary behaviour. The results of this study indicate, that health interventions targeting to reduce ST may improve it, if the focus would not only be SCT or media use. To confirm this assumption, further and deeper research is necessary. Sedentary behaviour should be investigated separately for the evaluated groups, as they seem to have different reasons for high ST. ST was only found to be related with SCT in children with migration background. Among overweight or obese children this study only found a tendency that ST is linked to SCT. Further studies need to clarify the potential reasons for overestimation of SCT by parents and misclassification of activity level among overweight and obese children. Finally, as girls spend more time being sedentary than boys but had the same duration of SCT, other activities also need to be considered especially among this group. Consequently, future studies should also investigate which activities children conduct during their sedentary time. Moreover, the role of possible compensation mechanisms should be considered in future research and when trying to intervene on ST.

## Abbreviations

Kg: Kilogram
LPA: Light physical activity
m^2^: Square meter
MB: Migration background
MVPA: Moderate to vigorous physical activity
NW: Normal weight
OW: Overweight/obese
PCT: Percentage of screen time on sedentary time
SCT: Screen time
ST: Sedentary time
UW: Underweight
